# Particle Debris Generated from Passenger Tires Induces Morphological and Gene Expression Alterations in the Macrophages Cell Line RAW 264.7

**DOI:** 10.3390/nano13040756

**Published:** 2023-02-17

**Authors:** Anna Poma, Massimo Aloisi, Antonella Bonfigli, Sabrina Colafarina, Osvaldo Zarivi, Pierpaolo Aimola, Giulia Vecchiotti, Lorenzo Arrizza, Alessandra Di Cola, Patrizia Cesare

**Affiliations:** 1 Department of Life, Health and Environmental Sciences, University of L’Aquila, 67100 L’Aquila, Italy; 2 Center for Microscopy, University of L’Aquila, 67100 L’Aquila, Italy; 3 Tun Abdul Razak Research Centre, Brickendonbury, Hertford SG13 8NL, UK

**Keywords:** macrophages, RAW 264.7, passenger tire microparticles, genotoxicity, comet assay, p53, p21, bax, Bcl-2, caspase 3, caspase 9

## Abstract

Air pollution in the urban environment is a topical subject. Aero-suspended particles can cause respiratory diseases in humans, ranging from inflammation to asthma and cancer. One of the components that is most prevalent in particulate matter (PM) in urban areas is the set of tire microparticles (1–20 μm) and nanoparticles (<1 μm) that are formed due to the friction of wheels with asphalt and are increased in slow-moving areas that involve a lot of braking actions. In this work, we studied the effect that microparticles generated from passenger tires (PTWP, passenger tire wear particles) have in vitro on murine macrophages cells RAW 264.7 at two concentrations of 25 and 100 μg/mL, for 24 and 48 h. In addition to the chemical characterization of the material and morphological characterization of the treated cells by transmission electron microscopy, gene expression analysis with RT-PCR and active protein analysis with Western blotting were performed. Growth curves were obtained, and the genotoxic effect was evaluated with a comet assay. The results indicate that initially, an induction of the apoptotic process is observable, but this is subsequently reversed by Bcl2. No genotoxic damage is present, but mild cellular abnormalities were observed in the treated cells.

## 1. Introduction 

Air pollution is the phenomenon of an increase of one or more molecules in the atmosphere beyond certain limits [1]. The increase in pollutants, in fact, has an impact first on the entire ecosystem: some molecules can alter atmospheric balances, such as chlorofluorocarbons, and contribute, for example, to the greenhouse effect and climate change [2,3]. Molecules suspended in air form atmospheric PM, composed of both organic and inorganic molecules, and are classified according to their size. They are referred to as PM10 if their aerodynamic diameter is smaller than 10, PM2.5 if their size is smaller than 2.5, “fine” if between 1 and 10 nm, and “ultrafine” if less than 10 nm [4]. The sources of such PM can be primary or secondary. Primary sources are those that are released into the environment by humans or by natural events and they are in their final form, while secondary are the reaction product of molecules in the air [5]. Anthropogenic sources can be highly variable (sulfur dioxide, ammonia, oxides of nitrogen) and associated with industrial activities and vehicle emissions [6]. Natural sources, on the other hand, are volcanic eruptions, fires, emissions from living organisms, and dust storms [7]. Among the main sources of air pollution are vehicles, particularly gaseous exhaust emissions [8]. In the last two decades, there has been a 22% reduction in PM10 concentrations, with peak pollution occurring in Southeast Asia [9]. These types of emissions are called non-exhaust emissions and are characterized precisely by the tire residues formed by friction on asphalt [10].

The main components of tires are SBR styrene rubber, butadiene rubber, natural rubber, organic peroxides, nitro compounds, selenium zinc, and other metals [11,12]. The mechanisms by which they are formed are mainly thermal in nature, i.e., due to friction with asphalt on roads. The shape and size of the fragments also determine the size of the PM that is formed [13]. It is precisely because of these plastic properties that some authors place them within the category of microplastics [14].

The human respiratory tract is composed of ducts that become thinner in diameter as one approaches toward the lungs. Consequently, smaller particles are the only ones capable of reaching the alveoli, thus causing toxic effects in the lungs [15]. Specifically, from the pharynx to the trachea, only a PM smaller than 4.7 μm passes, while in the alveoli, only that which is smaller than 0.65 μm arrives [15,16]. Such particles can pass through the lung epithelium and be transported in the blood to other organs as well [17]. The main effect on cells would seem to be the formation of reactive oxygen species (ROS) and oxidative stress. One hypothesis is that the metals associated with microparticles are the main contributors to these toxic effects [18,19]. In addition, genotoxic damage has been seen with an increase in micronucleus formation and TNF-alpha release from lung macrophages, especially when in the presence of common vehicle residues rather than truck residues [20]. This damage can cause chronic inflammation and cancer, making PM responsible for serious health consequences [21,22]. Kreider at al. [23] proposed a risk assessment by calculating the NOAEC (no observable adverse effect concentration) for humans by adjusting it according to exposure times. The result was 20 μg/m^3^.

ROS observed in vitro appear to be responsible for toxicity in vivo as well. In fact, they are associated with genotoxic damage and carcinogenesis [24,25,26]. An exposure to traffic pollution products can induce childhood asthma, with a higher risk for PM2.5 precisely because of its small size [27]. In addition, the increased induction of allergies has been seen in more exposed children [28]. Cardiac damage and myocardial hypertrophy with an overexpression of mir-208b/β-MHC has been observed in rats [29]. Additionally, at the epigenomic level, the increased acetylation of histone GATA4 has occurred [30].

In general, regarding rubber residues, no chronic toxicity has been observed on algae, *Daphnia magna*, and fish using sediment-extracted debris [31,32]. Some acute effects have been observed on the macroalga *Ulva lactuca* and on amphibians [4,33].

The toxicity of rubbers must also be studied in consideration of the chemicals they release once dispersed and the physicochemical conditions of the environment in which they are found. Indeed, pH, salinity, UV, and heat can determine what is released from such fragments [34]. In particular, biocides, flame retardants, plasticizers, pigments, antioxidants, and metals are released [35]. Consequently, the extraction processes for these materials are very complex and involve different protocols to maximize extraction [36]. According to the type of study, one must put in place the most suitable extraction protocol in order to eliminate interference from other components or leave it in case of studies on mixtures of pollutants [37,38]. This latter leads mainly to additional toxigenic effects in addition to those of the materials per se. Of interest are the two effects called “Trojan horse” and the “carrier effect” [39,40]. Therefore, in planning the study of microtire particles toxicity, one must necessarily take into consideration the effects that the accessory contaminants have since they have variously been seen to be determinants in the adverse effect [41]. In this work, the effect of the rubber particles of passenger tire wear particles rubber particles (PTWP) (PM10, smaller than or equal to 10 μm) on a macrophage cell line RAW 264.7 (RAW) was tested; the proliferation and viability, genotoxic effect, morphological changes, and variation in the gene expression and in the protein levels of the proteins involved in the stress response were evaluated.

## 2. Materials and Methods

### 2.1. Preparation of the Tire Rubber Sample

The samples used in this study were obtained from passenger tires and were acquired from the “Tun Abdul Razak Research Centre (TARRC) UK” research center. The samples were made artificially, recreating the phenomenon of abrasion between the tire and a road simulator device. “Passenger” tires are prone to quick wear; we have reproduced this phenomenon. The rubber debris were collected and analyzed further.

To obtain tire dust, we used Tissuelyser II (Qiagen) and Andersen impactor: the rubber debris were pre-cooled for 24 h at −80 °C and centrifuged in 3 cycles of 2 min each at a frequency of 25 Hz in the presence of 2 tungsten beads. With this procedure, we obtained a fine and homogeneous rubber dust, with most of the particles being smaller than or equal to 10 μm.

The rubber pulverized samples were sieved through the Andersen impactor to select the particles according to their size. An eight-stage Andersen cascade impactor [27] with a preparator stage allowed us to eliminate particles with aerodynamic diameters >10 mm.

The Andersen impactor sorts the finest particles similarly to the human respiratory system which, in turn, can be regarded as a PM dimensional classifier where only the finest, dangerous particles are able to pass the upper filtering districts and reach the alveolar region.

The operation of fractioning to the impactor was then repeated further by inserting an adhesive stab at the first stage. Through this stab, we recovered a small amount of rubber that was analyzed by SEM for the rubber particle morphological analysis and microanalysis of the metallic content of our samples.

### 2.2. Cell Culture

Raw 264.7 cells derived from mouse leukemic monocyte macrophages cultured from the ECACC (European Collection of Authenticated Cell Cultures provided by Sigma, Setagaya City, Tokyo, 85062803) were cultured as the monolayer in DMEM containing 10% (*v/v*) fetal bovine serum, 2 mM of L-glutamine, penicillin (100 IU/mL), and streptomycin (100 μg/mL) and they were maintained at 37 °C in a humidified atmosphere (95%) with 5% CO_2_.

The cells were seeded at approximately 15,000 cells/cm^2^ and sub-cultured when approximately 90% confluence was reached, detaching them from the plates with a 0.05% trypsin-0.02% EDTA solution (all cell material was purchased from Sigma Aldrich, St. Louis, MO, USA). The treatment with rubber powder was carried out 24 h after seeding. In all experiments, prior to cell treatment, rubber particle suspensions were subjected to 20 min in an ultrasonic bath to improve the homogeneous dispersion of the particles in the medium.

### 2.3. Cell viability and Growth

To evaluate the effect of rubber powder on the viability and cell growth, growth curves were prepared by seeding at a density of 15,000 cells/cm^2^ in a 6 multi-well plate (35 mm diameter). After 24 h, the treatment with different concentrations of rubber powder (25 μg/mL, 100 μg/mL) was carried out at the established times in a humidified incubator in a controlled atmosphere (5 % CO2, 90% humidity, 37 °C). After 0, 24, 48, and 72 h, the cells were counted by the Burker chamber with the dye exclusion Trypan blue method, diluted 1:10. Each sample was evaluated in triplicate.

### 2.4. Comet Assay

The alkaline comet assay was performed according to Singh et al. [42] and Collins et al. [43] using Trevigen’s comet slide (2 wells).

RAW cells treated or untreated (control) with different concentrations of rubber powder (25 μg/mL, 100 μg/mL) for 48 h were harvested and resuspended in PBS (1.5 × 10^5^ cells/mL); the positive control was carried out by treating the control cells with 100 μM of H_2_O_2_ for 30 min at 4 °C.

In total, 50 μL of the cells was added to 500 μL of molten LM Agarose (0.5% low-melting agarose), kept at 40 °C, and 50 μL of aliquot (750 cells) was pipetted onto an area of the comet slide. The slide was incubated at 4 °C for 10 min and then transferred to pre-cooled lysis solution (2.5 M NaCl, 100 mM EDTA, 10 mM Tris-base, 0.01% Triton X-100, 10% DMSO) for 60 min at 4 °C in the dark.

The slides were placed in an electrophoretic cell in alkaline buffer solution, prepared at the time of use (300 mM NaOH and 1 mM EDTA, pH 13), incubated for 20 min, then subjected to electrophoresis for 30 min, at 1V/cm; all operations were carried out in an ice bath and in the dark. Subsequently, the slides were neutralized with 0.4 M Tris, pH 7.5 in 3 successive washes of 5 min each, then immersed in 70% ethanol at room temperature for 15 min, air dried, and stored at 5 °C. Two biological replicates and two technical replicates were performed. DNA was stained with ethidium bromide (2 µg/mL) for 5 min. The slides were observed by epifluorescence microscopy with a 490 nm filter; 7 fields were acquired for each well using ZeissAxio Imager M2 microscopy, and the coordinates of the fields were the same for each slide. For each condition, images of 200 randomly selected cells (50 cells from each well) were analyzed with CASP Lab software.

The statistical evaluation was performed using the two-tailed Student’s *t*-test. A difference at *p* < 0.05 was considered significant.

### 2.5. Gene Expression Analysis

Variations in the gene expression in RAW cells treated or untreated (control) with different concentrations of rubber powder (25 μg/mL, 100 μg/mL) for 24 and 48 h were evaluated by qRT-PCR.

The genes analyzed were: BCL2-associated X protein (Bax, ENSMUSG00000003873), Caspase 3 (Casp3, ENSMUSG00000031628), Caspase 9 (Casp9, ENSMUSG00000028914), B cell leukemia/lymphoma 2 (Bcl-2, ENSMUSG00000057329), cyclin-dependent kinase inhibitor 1 (p21, ENSMUSG00000023067), transformation-related protein 53 (Trp53, p53, ENSMUSG00000059552), and glyceraldehyde-3-phosphate dehydrogenase (Gapdh, ENSMUSG00000057666). The primers mix 20× were provided by Bio Rad; Table 1 shows the identification code of the primer pair. The expression levels of Gapdh were measured as a reference level of the target gene expression.

Total RNA was isolated from RAW cells using Trizol reagent (Invitrogen) according to the manufacturer’s instructions. The RNA concentration was assessed by Qubit Fluorometers (Thermo Fisher Scientific, Waltham, MA, USA).

First-strand cDNA synthesis was performed from 1 µg of total RNA using an iScript^TM^ cDNA Synthesis Kit (Bio-Rad) according to the manufacturer’s protocol; at the same time, a no-RT control reaction was carried out. qRT-PCR was performed in a final volume of 20 µL, including 25 ng of the cDNA product, 1 µL of specific forward and reverse primer, and 10 µL of 2 × SsoAdvanced Universal SYBR Green Supermix (Bio-Rad) according to the manufacturer’s protocol.

The relative expression ratio between the treated and non-treated cells was analyzed using a delta–delta Ct method (2^−ΔΔCt^). Two biological replicates were performed, and all the samples were carried out in duplicate.

### 2.6. Western Immunoblot Analysis

The protein content variation in the RAW cells treated or untreated (control) with different concentrations of rubber powder (25 μg/mL, 100 μg/mL) for 24 and 48 h was evaluated by Western immunoblot analysis.

The cells (control and treated) were collected, and a total protein extraction was performed with RIPA lysis buffer (Cell Signaling Technology, Inc., Danvers, MA, USA) containing protease inhibitors (1 mM phenylmethyl sulphonyl fluoride, 1 µg/mL aprotinin, 1 µg/mL leupeptin) and phosphatase inhibitors (10 mM sodium pyrophosphate, 1 mM sodium fluoride, and 1 mM sodium orthovanadate). The samples were processed by mechanical lysis with the Gilson pipette (10 times up and down). After centrifugation (14,000 rpm for 10 min at 4 °C), the supernatants were used for the protein separation by electrophoresis SDS-PAGE, according to Laemmli [44,45], and the cell extracts were quantified using the BCA™ Protein Assay (Thermo Scientific, Thermo Fisher Scientific Inc.). The samples were then diluted in the sample buffer (200 mM Tris-HCl pH 6.8, 40% glycerol, 20% β-mercaptoethanol, 4% sodium dodecyl sulphate, and bromophenol blue) and 30 μg of the proteins were loaded and separated on gel 12% in a running buffer at 200 V for 60 min and transferred to PVDF membranes (Sigma Aldrich) using a mini electrophoretic cell transfer (Bio-Rad Laboratories, Hercules, CA, USA). The membranes were blocked in TBST with 5% *w/v* non-fat dry milk for 60 min and incubated with the appropriate primary and secondary antibodies.

The membranes were incubated with the subsequent primary antibodies at 4 °C overnight: anti-Casp3 specific for the active form (1:500), anti-Casp9 specific for the active and inactive form (1:500), anti-Bax (1:200), anti-Bcl-2 (1:200), anti-p21 (1:500), anti-p53 (1:1000), and HRP-linked anti-rabbit IgG (1:1000), and HRP-linked anti-mouse IgG (1:1000) were used as secondary antibodies (2 h, room temperature); the peroxidase activity was detected using an ECL West Pico Plus Substrate, the UK. The densitometric quantification was performed by the Q9 Alliance imaging system (Uvitec, Cambridge). β-actin (1:2000) were used as internal controls for equal protein loading and the values were given as relative units (RU). Images of the bands were analyzed by using Nonlinear Dynamics TotalLab software (TotalLab Ltd., Newcastle upon Tyne, the UK). Western blotting was performed in duplicate for at least three independent experiments. The antibodies purchased were anti-Casp9, anti-p53, and anti-Bax from Santa Cruz Biotechnology, Inc. Santa Cruz, CA, USA, anti-Casp3 from Cell Signaling Technology, Inc., Danvers, MA, USA, anti-Bcl-2 and anti-p21 from Proteintech Europe, Manchester, the UK, and the secondary antibody from Immunological Sciences, Rome, Italy.

### 2.7. Analysis of the Tire Rubber Particles by SEM and EDS Analysis

For the morphological analysis and the microanalysis of passenger rubber particles, we used the SEM Zeiss Gemini 500 equipped with EDS (energy dispersive spectroscopy) Oxford AZtec Live, powered by Ultim Max 100. The purpose of EDS microanalysis is to provide specific information on the composition of the sample elements.

We inserted a carbon tape in the zero stage of the Andersen impactor to recover sufficient quantities of the passenger tires. The carbon tape was mounted onto an SEM stub and the sample was observed working under controlled pressure (23 Pa) without being coated with a thin gold film by the sputtering method.

The SEM observations were carried out at different magnifications, and morphological analysis of the particles was performed simultaneously to obtain the EDS microanalysis of selected particles.

### 2.8. TEM Analysis of RAW Cells

For ultrastructural analysis by transmission electron microscopy, RAW cells, treated (100 µg/mL for 24 and 48 h) and the control, were preliminarily embedded in Durcupan ACM epoxy resin; after being detached and collected from culture flasks, they were fixed with 2.5% glutaraldehyde in PBS (1 h, 4 °C) and subsequently post-fixed with 1% osmium tetroxide in 0.1 M pf cacodylate buffer, pH 7.2, for 1 h at 4 °C. Then, they were dehydrated in an ethanol/propylene oxide series and embedded in Durcupan ACM epoxy resin, and finally cut with a Sorvall Porter Blum MT2-B ultramicrotome. Ultrathin sections (70 nm thick) were collected onto copper grids and double-stained with 5% uranyl acetate in 70% ethanol and lead citrate (Reynolds). Observation and imaging were performed with a Philips CM100 transmission electron microscope at the Microscopy Center of the University of L’Aquila, Italy. (At least 50 cells were accurately examined for each sample analyzed.)

### 2.9. Statistical Analysis

Student’s *t* test (unpaired) was applied for the statistical analysis of the data obtained from the tests to verify if the mean value of the treated conditions differs significantly from a reference value (control). For statistically significant values, * = *p* < 0.05; ** = *p* < 0.005; *** = *p* < 0.0005.

The error bars represent the standard error of the mean. The data were analyzed using the GraphPad Prism software, version 6.0 (Ó 1995–2015 GraphPad Software, Inc.). Three independent experiments have been performed for all assays applied.

## 3. Results

### 3.1. Growth Curves

**Figure 1:**
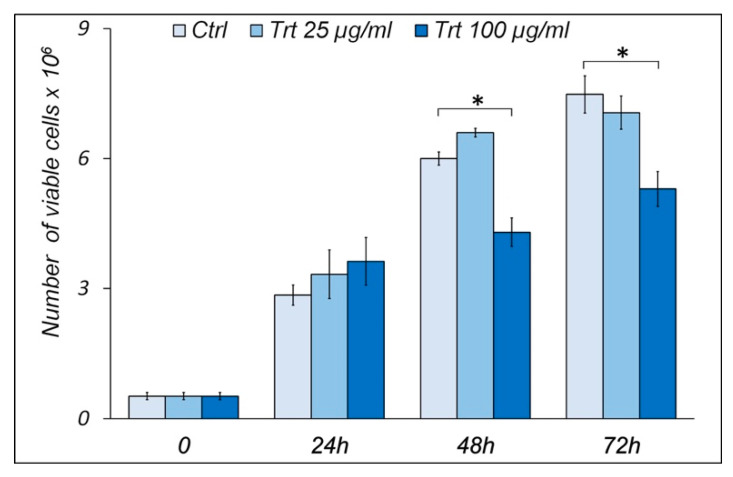
Effect of rubber powder on viability and cell growth. Histograms show number of viable RAW cells, treated (Trt) or untreated (Ctrl) for 24, 48, and 72 h with 25 or 100 μg/mL of passenger tire wear particles, evaluated by exclusion Trypan blue method. On the other hand, at 100 μg/mL, there is a reduction in cell proliferation both at 48 and at 72 h. No significant changes have been observed previously. Under all conditions, cells appear viable. Means ± S.E.M. are reported (n = 3). Student’s *t* test was employed to compare the values of untreated (control) and treated cells. * *p* < 0.05.

Figure 1 shows the growth curves of RAW cells treated or untreated (control) with different concentrations of rubber powder (25 μg/mL, 100 μg/mL) for 24, 48, and 72 h. The treated cells with 25 μg/mL of rubber did not show significant differences in cell proliferation compared to the control at any of the times considered.

### 3.2. Comet Test

**Figure 2:**
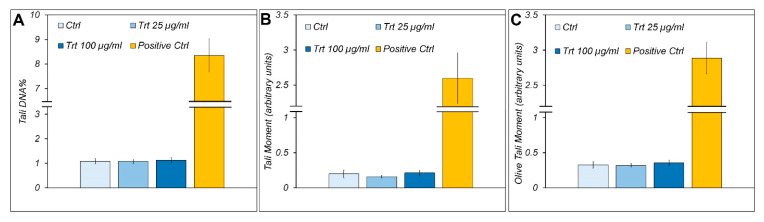
Alkaline comet assay. Evaluation of DNA damage by the alkaline comet assay in RAW cells, treated (Trt) or untreated (Ctrl) for 48 h with 25 or 100 μg/mL of passenger tire wear particles. Positive control = 100 μM H_2_O_2_ at 4 °C for 30 min. Histograms show means ± S.E.M (n = 200) of tail DNA % (**A**), tail moment (**B**), and olive tail moment (**C**).

The comet assay is used to test the clastogenic and genotoxic damage of rubber on the cells. The quantitative analysis for the alkaline comet assay showed no increased tail DNA % (Figure 2A), tail moment (Figure 2B), and olive tail moment (Figure 2C) after 48 h of treatment with 25 and 100 µg/mL of gum, indicating no significant DNA damage. These parameters increase up to 10 times in the positive control, which was carried out by treating the cells with H_2_O_2_, as described in the Materials and Methods. 

### 3.3. Gene Expression

**Figure 3:**
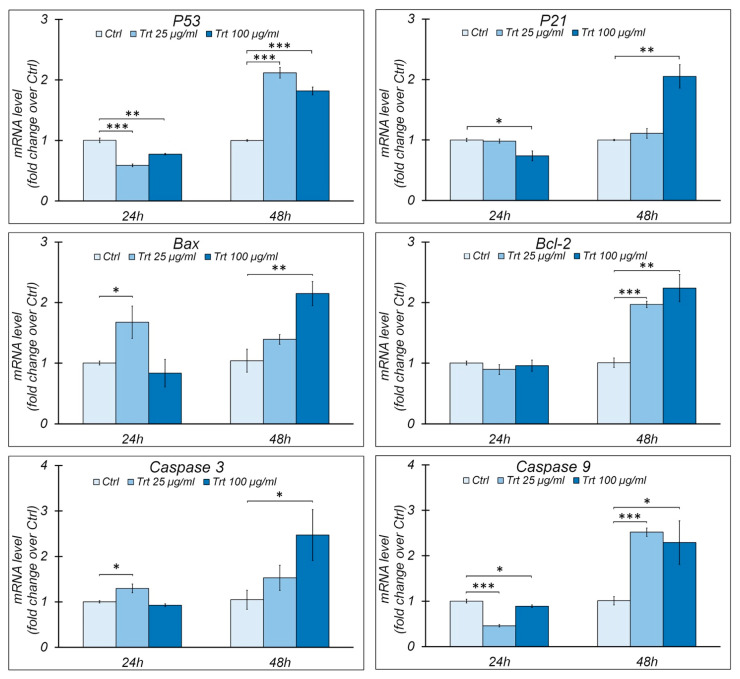
Gene expression by RT-qPCR. Analysis of gene expression by RT-qPCR of p53, p21, Bax, Bcl-2, caspase 3, and caspase 9 in RAW cells, treated (Trt) or not (Ctrl) for 24 or 48 h with 25 or 100 μg/mL of passenger tire wear particles. Histograms show the relative mRNA level, normalized to endogenous control Gapdh and expressed as fold change over control. Means ± S.E.M. are reported (n = 4). Student’s *t* test was employed to compare the values of untreated (control) and treated cells. * *p* < 0.05; ** *p* < 0.005; *** *p* < 0.0005.

In RAW cells, treated or untreated (control) with different concentrations of rubber powder (25 μg/mL, 100 μg/mL) for 24 and 48 h, the variation in the gene expression was evaluated for the cell cycle regulators p21 and p53 of the proapoptotic proteins Bax, caspase 3, caspase 9, and antiapoptotic proteins Bcl-2 by qRT-PCR (2^−ΔΔCt method^) (Figure 3).

p21, also known as p21waf1/cip, is a well-known inhibitor of cell cycle progression; it acts by inhibiting the activity of cyclin-dependent kinases, and p53 is its main transcriptional regulator.

As shown in Figure 3, the gene expression of p53 and p21 increases significantly only in the cells treated for 48 h; the transcript level of p53 increases with both concentrations of rubber powder, while the p21 expression increases only at the higher concentration.

As for the proteins involved in apoptosis (Figure 3), in general, an increase in transcripts compared to the controls is observed only after 48 h of treatment (with the exception of Bax, which also increases at 24 h).

Specifically, after 48 h of treatment, Bax and Caspase 3 increase only at the highest concentration, while Bcl-2 and Caspase 9 increase at both concentrations.

### 3.4. Immunoblot Analysis

**Figure 4:**
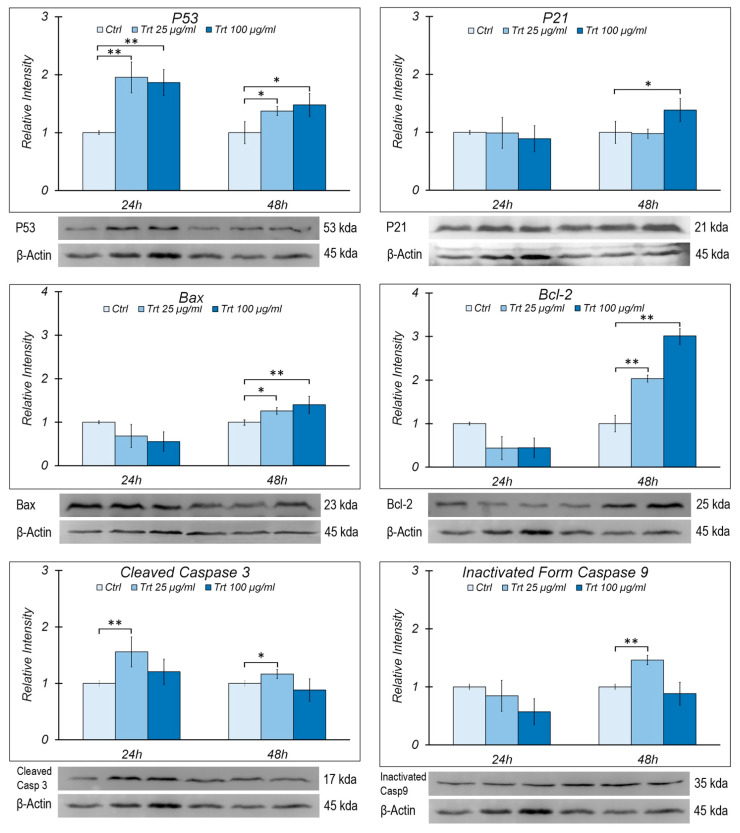
Western blots analysis of protein levels of p53, p21, Bax, Bcl-2, cleaved caspase 3, and inactive form of caspase 9 in RAW cells, treated (Trt) or untreated (Ctrl) for 24 or 48 h with 25 or 100 μg/mL of passenger tire wear particles. Histograms showing the relative intensity of the Western blot bands of these proteins along with the representative Western blots are shown. The densitometry values were normalized for β-actin and were compared with the control. The means ± S.E.M. are reported (n = 3). Student’s *t* test was employed to compare the values of the treated and non-treated cells. * *p* < 0.05; ** *p* < 0.005.

In RAW cells, treated or untreated (control) with different concentrations of rubber powder (25 μg/mL, 100 μg/mL) for 24 and 48 h, the variation in the protein level was evaluated for the cell cycle regulators p21 and p53 of the proapoptotic proteins Bax, caspase 3, caspase 9, and antiapoptotic proteins Bcl-2 by immunoblot analysis (Figure 4).

The p53 and p21 protein levels tend to increase relative to the control. p53 increases in both concentrations, at 24 h and 48 h, showing how the cells perceive stress and implement response mechanisms; p21 is most expressed only at the highest concentration and after 48 h. The results are consistent with the cells’ proliferation, showing that only prolonged stress can induce a complex response; in particular, the cell responds by activating p53 which in turn activates p21, thus causing the cell cycle to stop. Additionally, with regard to Bax and Bcl-2, there are effects only at higher concentrations and at 48 h. In particular, Bax increases significantly to 100 µg/mL at 48 h, whereas Bcl-2 increases at 48 h at both concentrations (25 and 100 µg/mL).

In regard to caspase 9, only the inactive form was detected, which increases after 48 h of treatment with 25 µg/mL of rubber powder; the active form of caspase 3 (17 kda) increases in the treated cells for 24 and 48 h at the same rubber concentration.

### 3.5. Analysis of the Tire Rubber Particles by SEM and EDS Analysis

Passenger particle samples were observed with scanning electron microscopy (SEM) equipped with EDS for elemental analysis. The dimensions of the particles range from 9 to 10 microns, and they tend to aggregate.

**Figure 5:**
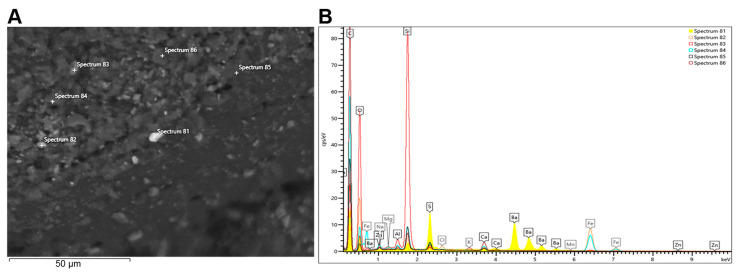
Scanning electron microscopy (SEM). Analysis of passenger particles (**A**) and the related spectrum of the microanalysis with EDS (**B**).

Some significant particle samples, recovered at the first stage of the impactor, of which the morphological images are reported, were analyzed (Figure 5A).

In addition to the morphological study, an elemental composition analysis was carried out using the EDS analysis to obtain an X-ray emission spectrum for the particles. In the spectrum of the particles, different peaks corresponding to carbon and silica, followed by oxygen, may be observed; traces of sodium, barium, iron, aluminum, magnesium, calcium, and zinc are also present (Figure 5B).

### 3.6. TEM (Transmission Electron Microscopy) Analysis of RAW Cells

**Figure 6:**
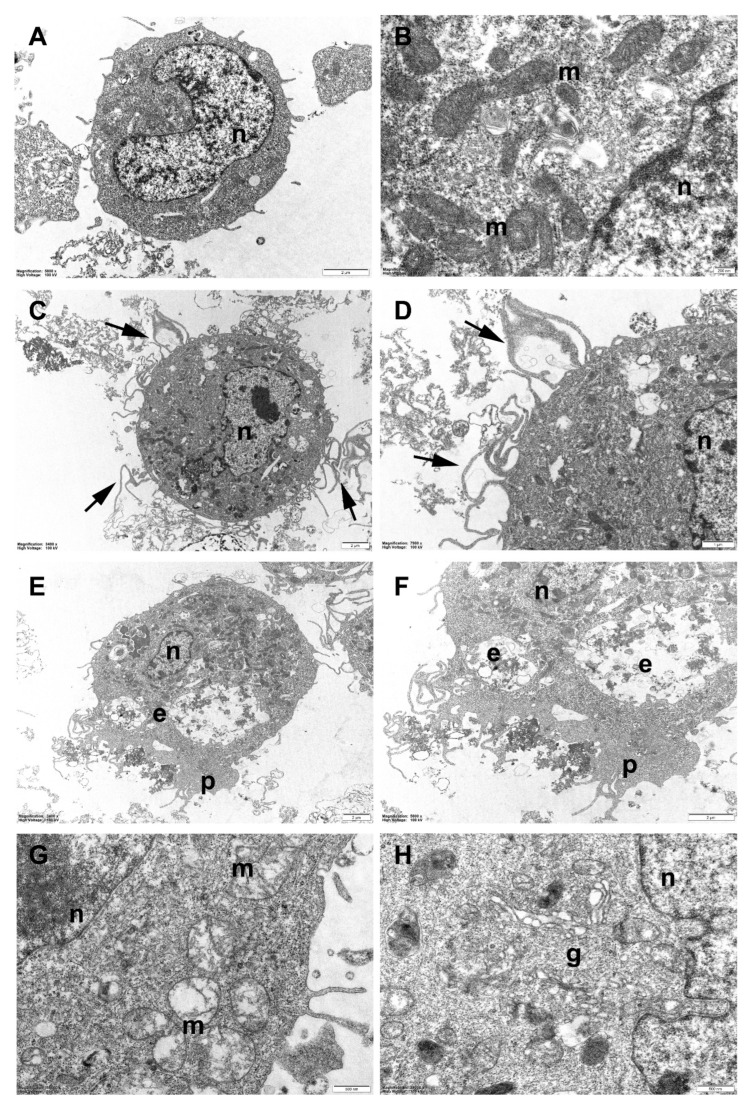
Transmission electron microscopy (TEM). Representative TEM images of RAW264.7 cells after incubation with passenger tire wear particles for different time periods. (**A**,**B**) unexposed (control) cells; (**C**–**F**) cells incubated for 24 h; (**G**,**H**) cells incubated for 48 h. Note, after 24 h incubation, signs of macrophage activation: more abundant and elongated filopodia (arrows), particularly evident in (**C**,**D**) (detail); increased phagocytic activity, well visible in (**E**,**F**) (at higher magnification). After 48 h of incubation, mitochondria (**m**) appear mostly swollen and structurally deranged ((**G**), compare with normal mitochondria in control cells, (**B**)) and disorganized Golgi apparatus (**g**) is sometimes observed (**H**). **e**, endocytic vesicles; **n**, cell nucleus; **p**, pseudopodium.

Untreated (control) RAW cells exhibited the typical morphological features of cultured alveolar macrophages (Figure 6A). They appeared mostly round and displayed some (generally) short plasma membrane protrusions (filopodia and/or pseudopodia) extending from their cell surfaces. Their nuclei were relatively large, with a more or less regular outline, and numerous mitochondria, vesicles, and lysosomes were visible in the rather electron-dense cytoplasm (Figure 6B).

After incubation with rubber powder for 24 h, the RAW cells underwent morphological changes indicative of (macrophage) activation. Longer and more numerous filopodia could be seen on their surface, a sign of increased mobility (Figure 6C,D), while more prominent pseudopodia associated with plasma membrane invaginations and abundant endocytic vesicles were frequently observed, suggesting an increase in the phagocytic activity (Figure 6E,F). Furthermore, treated cells exhibited a clearly evident decrease in the nucleus/cytoplasm ratio, especially when more conspicuous signs of activation were present. On the other hand, no specific structural alterations in the cell organelles or evidence of cytotoxic damage could be detected.

On the contrary, 48 h of incubation with rubber powder seems to be able to cause some harmful effects on RAW cells. Morphological alterations were particularly evident in mitochondria, that appeared frequently swollen and structurally deranged, with altered cristae and a less dense mitochondrial matrix (Figure 6G). In some cases, a certain degree of disorganization of the Golgi apparatus could also be seen (Figure 6H).

## 4. Discussion

In this work, the effect of rubber particles (PM10, smaller than or equal to 10 μm) on a macrophage cell line (RAW) was tested; the proliferation and viability, genotoxic effect, morphological changes, and variation in the gene expression and in the protein levels of the proteins involved in the stress response were evaluated.

After treatment with rubber powder, no genotoxic or necrotic effect is observed, as evidenced by the results of the Comet test and trypan blue, respectively. The proliferation curves show that only the highest concentration 100 µg/mL and prolonged stress (48 and 72 h) are able to influence the cell proliferation; this result is consistent with chronic rather than acute toxicity.

Treatment with rubber particles at both concentrations causes an increase in p53, indicating a stressful stimulus; the gene expression increases at 48 h, while the protein content increases both at 24 and 48 h, suggesting a slow turnover of p53 [46].

p53 is the main transcriptional regulator of a p21 known cell cycle inhibitor; p21 increases both in the gene expression and in the protein content at 48 h of treatment and at the highest concentration, which is consistent with the decreased proliferation observed with the growth curve.

At 24 h, for both rubber powder concentrations, the Bax/Bcl-2 ratio increases with treatment, indicating that the cells are preparing to initiate the apoptotic process; at 48 h, with regard to the pro-apoptotic proteins Bax, caspase 3, and 9, these increase with the treatment both in the gene and protein expression; however, the increase in the expression and protein content of Bcl-2 and the decrease in the Bax/Bcl-2 ratio for both concentrations indicate a prevalence of the mechanism of inhibition of apoptosis. On the other hand, caspase 9 is not activated. The reduced effect is likely due to the antiapoptotic action of Bcl-2, which increases over time, thus reducing the expression of caspases at prolonged times and at higher concentrations.

The inhibition of apoptosis observed by us could also be caused by the increase in p21 as other important functions have been attributed to p21, such as the transcriptional regulation and modulation/inhibition of apoptosis [47].

The presence of the active form of caspase 3 at the lowest concentration for both times could indicate that an additional apoptotic pathway is involved in the response to treatment.

In this work, we conducted a “point analysis” of the main components of the particles. The particles were characterized by size and morphology with SEM; we obtained the chemical characterization of the elements in a sample volume using energy dispersive X-ray spectroscopy (EDS). Further analyses of these types of particles are needed to find a more stringent correlation with the toxicity associated with the particle debris generated from passenger tires in outdoor samples. As an example, in the study reported in [48], the authors estimated PAH emissions from biomass burning in cooking stoves in three Chinese provinces using the measured real-world EFs (emission factors); this study was made possible by taking the spatial difference into consideration and synthetically coupling real-world EF tests and an emission inventory compilation. In another context [49], the author examines non-exhaust PM emissions from tires, as well as brake and road wear, which have become the dominant sources of vehicle-derived PM; this work suggests that studies on non-exhaust PM emissions from tire/brake/road wear have become essential as they contribute to the current debate around battery electric vehicles (BEVs) as non-emitting vehicles. In conclusion, the relevance of our work is to study particle debris generated from passenger tires as one of the main sources of vehicle-derived PM. Understanding the in vitro cyto/genotoxic effects and gene expression alterations by these particles is essential to the investigation of the effects of tire/microplastics particles on exposed organisms both in vitro and in vivo.

## Figures and Tables

**Table 1:** Bio-Rad IDs for the qRT-PCR primers.

Gene Name	Primers Bio Rad Unique Assay Id
Bax	qMmuCID0006274
Casp3	qMmuCID0005880
Casp9	qMmuCID0005382
Bcl-2	qMmuCED0039968
p21	qMmuCED0025027
p53	qMmuCID0006264
Gapdh	qMmuCED0027497

## Data Availability

All data included in this study are available upon request from the corresponding author.
